# The use of point-of-care ultrasound in a regional emergency department in KwaZulu-Natal, South Africa

**DOI:** 10.4102/safp.v63i1.5269

**Published:** 2021-08-23

**Authors:** Halalisiwe B. Khanyi, Bavani Naicker

**Affiliations:** ^1^Division of Emergency Medicine, School of Clinical Medicine, University of KwaZulu-Natal, Durban, South Africa

**Keywords:** point-of-care ultrasound, emergency ultrasound, emergency care, emergency department, emergency medicine, critical care, primary care, prehospital care

## Abstract

**Background:**

Formal ultrasonography has advanced to point-of-care ultrasound (POCUS) in the emergency department (ED) for the purpose of acute critical care. While POCUS application expands, little is known about POCUS utilisation in public hospital EDs. This study aimed to describe the use of POCUS in an ED in KwaZulu-Natal.

**Methods:**

A retrospective chart review study was conducted on all patients who had POCUS exams performed in the ED at the General Justice Gizenga Mpanza Regional Hospital from 01 September 2019 to 31 March 2020. A data collection tool was used to extract the required data from the Mindray M6 ultrasound machine. The data were processed using the Statistical Package for Social Sciences (SPSS version 26) and descriptive statistics were used to summarise the data.

**Results:**

A total of 978 POCUS were performed on 784 patients. Point-of-care ultrasound was utilised more often for focused emergency echocardiography in resuscitation (*n* = 383) and extended focused assessment with sonography for trauma (*n* = 319). The findings were normal in 17% of exams, 31% were positive, 9% were unspecified and 43% of POCUS exams were inconclusive. Seven percent of POCUS exams were performed by accredited level 1 emergency POCUS providers and ultrasounds occurred more frequently during day-shift hours than after-hours.

**Conclusion:**

Point-of-care ultrasound core applications were utilised by ED doctors for various emergency care scenarios, mainly for trauma and cardiac assessments.

## Introduction

The burden of trauma and medical diseases in South Africa is high.^1,2^ Public hospital emergency departments (EDs) are the first point of contact for various medical and trauma emergencies, with many of these healthcare facilities limited to plain radiography services. Although resource limitations persist, there has been an increase in the number of emergency medicine specialists in the EDs since the introduction of the specialty in 2003 and an advance in accredited level 1 emergency point-of-care ultrasound (POCUS) providers in the country.

Ultrasound is a safe, repeatable, non-invasive and cost-effective diagnostic tool. Point-of-care ultrasound is practiced in austere environments for applications related, but not limited, to battlefield triage, in-flight examination of critically ill transfers and screening at high altitudes for high-altitude emergencies; additionally, in-hospital facilities such as the operating theatre (OT) and EDs use POCUS.^3^ In the ED, POCUS has shown to be quick, focused, goal-directed with minimal delay or need for specialised technical personnel. Point-of-care ultrasound has been well-established, validated protocols for use in acute emergencies.

The integration of the extended-focused assessment with sonography for trauma (e-FAST) in the initial assessment for suspected thoraco-abdominal injury has shown to have a therapeutic impact in patients with severe trauma, and the accuracy of lung ultrasound has also shown to be superior to that of chest radiography in the detection of a pneumothorax.^4,5^ The use of focused cardiac ultrasound (FOCUS) has aided in diagnostic assessments, monitoring, therapy titration and procedural guidance in critically ill patients at the bedside.^6^ While studies have emerged noting potential harm of POCUS in prolonging cardiopulmonary resuscitation (CPR) pause duration, advances in resuscitation strategies during cardiac arrest and following return of spontaneous circulation (ROSC) have incorporated POCUS as a tool for detection of reversible causes of pulseless electrical activity (PEA).^7,8,9^

Retrospective analyses have demonstrated the ability of emergency physicians to perform colour Doppler ultrasound and reduce time-to-disposition of patients while also maintaining good correlation in results during screening for deep vein thrombosis (DVT)^10,11^; additionally, a bedside abdominal aortic aneurysm ultrasound performed by an emergency physician has shown to be accurate and fast.^12^ Emergency physicians have also demonstrated proficiency in evaluating patients at risk of ectopic pregnancy, detecting hydronephrosis in suspected ureteral colic and diagnosing ocular pathology in ED.^13,14,15,16^

The traditional paradigm of ultrasonography being performed by radiologists may become replaced by the POCUS paradigm in the ED as more methods of POCUS utility are described and published for acute critical care.

### Aim of study

The purpose of this study was to describe the use of POCUS in an ED in KwaZulu-Natal, South Africa.

### Specific objectives

- To measure the number of POCUS exams performed from 01 September 2019 to 31 March 2020.
- To determine the types of POCUS exams performed.
- To evaluate the frequency of each type of POCUS exam performed.

## Research methods and design

### Profile of the study location and population

The study was conducted at General Justice Gizenga Mpanza Regional Hospital (GJGMRH) which is the only regional hospital in the public sector in the Ilembe district, located 47 km from King Shaka International Airport. To date, the Ilembe district has over 600 000 residents with 55% of people living below the poverty line, 26.6% unemployed and only 7.9% of the population with medical aid coverage.^17^ The ED at GJGMRH receives direct referrals from the nine district clinics, the community health centres, district hospitals and general practitioners and accepts self-referrals.

### Study design and setting

This study was a retrospective chart review of POCUS performed in the ED at GJGMRH from 01 September 2019 to 31 March 2020.

### Study population and sampling strategy

This was a purposive sampling strategy of all patients in the ED at GJGMRH that had POCUS exams performed using the Mindray M6 ultrasound machine. As it was a descriptive study design, power analysis was not conducted.

### Inclusion criteria

All patients who had POCUS exams performed using the Mindray M6 Premium Portable Ultrasound were included for the study.

This model of ultrasound machine provided accurate information on frequency of use as it automatically captured date and time for every use.

### Exclusion criteria

- Patients who had POCUS exams performed using an alternative ultrasound machine were excluded, as necessary data could not be retrieved from that model of ultrasound machine.
- Patient hospital folders and paper-based POCUS reports were also excluded to reduce inconsistency in coding of chart information and missing charts.
- Paediatric patients with clinical presentations relating to medical causes, as these were assessed by the paediatric outpatient department (POPD).
- Point-of-care ultrasound exams performed using the Mindray M6 ultrasound before and after the study period.

### Data collection methods and procedure

The data were collected from the Mindray M6 ultrasound’s internal hard drive.

The iStationTM (Mindray’s Patient Information Management System) was used to retrieve, review and assess the ultrasound entries in chronological order.

A data collection tool was then used to collect the data needed for the study with all information fully anonymised with no key to the identity of the data subjects.

The data were collected by the primary investigator (an accredited level 1 emergency point-of-care ultrasound provider) who reviewed and analysed all saved POCUS records of still images (joint photographic experts group [JPEG] files), video clips (audio video interleave [AVI] files) and portable document format (PDF) reports for each study participant. In accordance with the American College of Emergency Physicians (ACEP) Emergency Ultrasound imaging criteria compendium,^18^ each POCUS exam type required the cardinal imaging planes (standard views) to be present to conclude the findings as normal, unless a PDF record of the POCUS exam was reported as normal. Point-of-care ultrasound exams that had inadequate standard views were reported to be inconclusive unless a positive finding was identified for that particular POCUS exam type. A second practitioner, certified as a level 1 emergency point-of-care ultrasound instructor, would review the records if any discrepancies in data collection or interpretation became apparent.

The data were collated by the primary investigator onto a Microsoft Office Excel spreadsheet and subsequently analysed by the University of KwaZulu-Natal College of Health Sciences Biostatistics Department, using the Statistical Package for Social Sciences (SPSS version 26). Descriptive statistics such as frequencies, proportions, mean and standard deviations were used to summarise the data.

### Ethical considerations

Ethical approval was granted by the University of KwaZulu-Natal Biostatistics Research Ethics Committee (BREC reference: BREC/00001281/2020) and the KwaZulu-Natal Department of Health (NHRD reference: KZ_202006_037).

## Results

**FIGURE 1:**
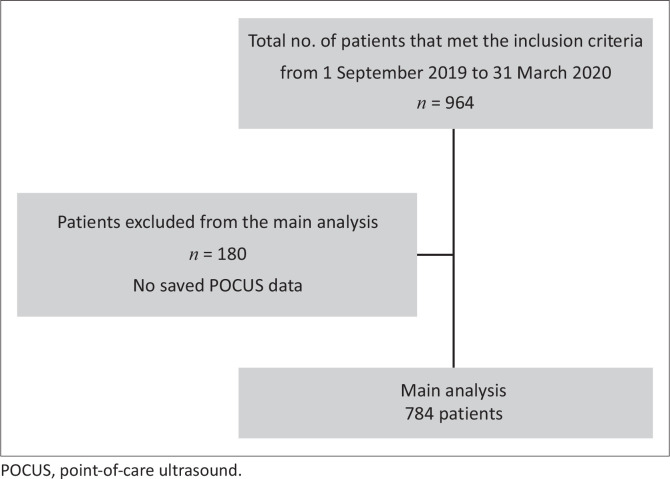
Flowchart of the sampling strategy.

During the study period, there was a total of 964 study participants who met the inclusion criteria. One hundred and eighty of those participants only had a record of time and date saved. These participants were subsequently excluded because they had no record of POCUS type(s), images, video clips or PDF data saved. Therefore, most of the data on POCUS were based on 784 study participants (Figure 1).

The mean age of the studied population was 42.89 with a standard deviation of ±20.62 years. The male population had more POCUS exams (54.04%) than that of the opposite gender (45.96% were female).

**FIGURE 2:**
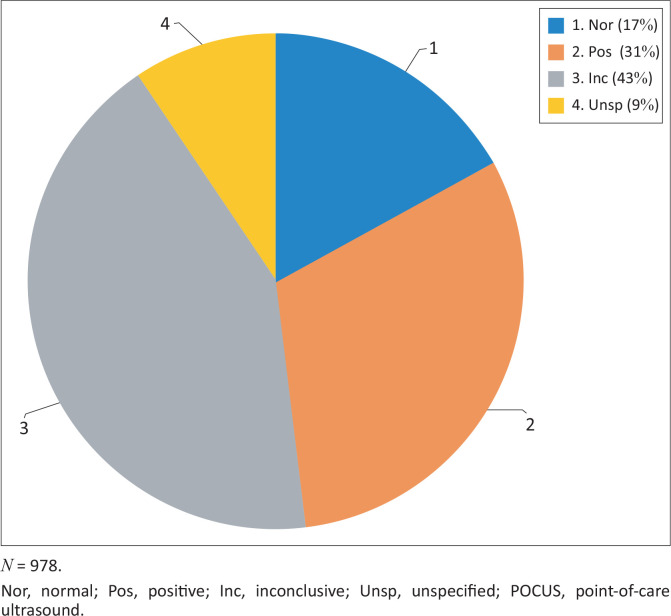
Total number of point-of-care ultrasounds performed and percentage contribution of the point-of-care ultrasounds findings.

From 01 September 2019 to 31 March 2020, a total of 978 POCUS exams were performed using the Mindray M6 ultrasound machine (Figure 2). The findings were normal in 17% of POCUS exams, positive in 31% of exams, 9% of exams were unspecified in terms of POCUS type and 43% of POCUS exams were inconclusive.

**FIGURE 3:**
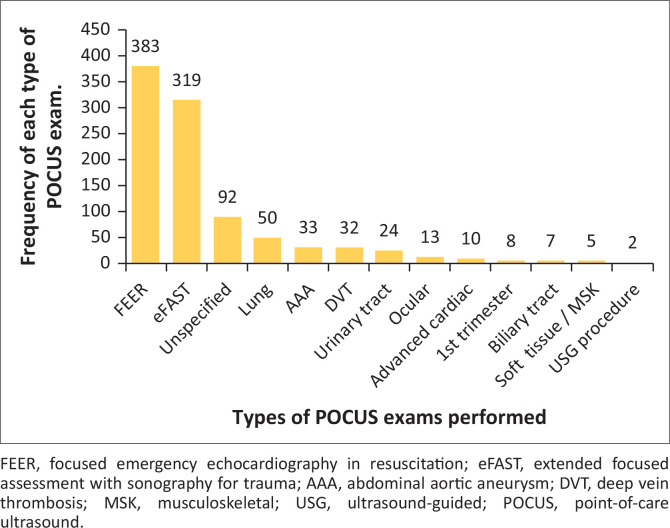
The types of point-of-care ultrasound exams performed and frequency of each type.

**FIGURE 4:**
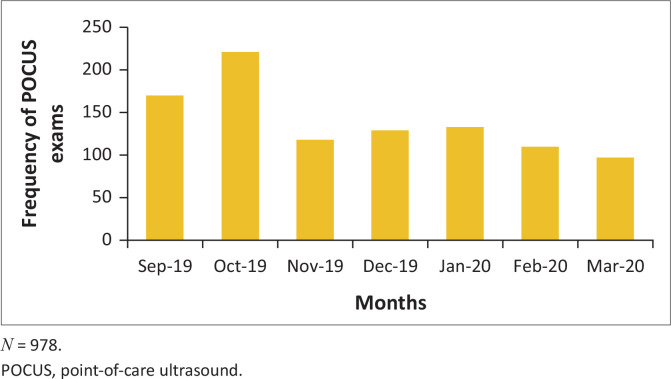
Frequency of point-of-care ultrasound exams performed per month.

Different types of POCUS were performed at variable frequencies (Figure 3). Point-of-care ultrasound was utilised more often for focused emergency echocardiography in resuscitation (FEER) exams and eFAST exams with the month of October having the highest number of POCUS exams performed (Figure 4).

Positive findings were detected with POCUS exams (Table 1). Pleural free fluid was the most common finding with positive eFAST exams. Mitral valve disease, dysrhythmia, dilated cardiomyopathy and congenital heart anomaly were some of the additional abnormalities detected with FEER POCUS. Hydronephrosis, cholecystitis and enlarged optic nerve sheath diameter were noted on POCUS; additionally, POCUS was utilised for DVT and abdominal aortic aneurysm (AAA) screening.

**TABLE 1:** Positive findings detected on point-of-care ultrasound listed by examination type.

Positive findings per POCUS exam type†	Positive findings detected (*n*)
**eFAST (*n* = 54)**	
Pleural free-fluid	24
Pneumothorax	17
Abdominal free-fluid	14
Pericardial free-fluid	8
**FEER (*n* = 173)**	
Flat IVC	77
RV dilatation	75
Other (VHD, dysrhythmia, DCMO, CHD)	71
Pericardial effusion	42
Absent wall motion	4
**DVT (*n* = 7)**	
Popliteal + femoral	3
Popliteal	2
Femoral	1
Not specified	1
**AAA (*n* = 5)**	
3.0 cm – 3.9 cm	3
4.0 cm – 4.9 cm	2
**USG procedure (*n* = 2)**	
Saline agitation test	1
Ascitic tap	1
**Biliary tract (*n* = 2)**	
GB stone	2
GB wall thickness	1
Sonographic Murphy’s	1
**Urinary tract (*n* = 10)**	
Hydronephrosis	7
Bladder mass	1
Bladder distension	1
Urolithiasis	1
**Ocular (*n* = 7)**	
ONSD > 5 mm	6
Globe rupture	1
**Lung (*n* = 37)**	
Pleural effusion	35
Pneumothorax	1
Post intubation reduced lung sliding	1
**Advanced cardiac (*n* = 6)**	
Impaired LV function	6

eFAST, extended focused assessment with sonography for trauma; FEER, focused emergency echocardiography in resuscitation; DVT, deep vein thrombosis; AAA, abdominal aortic aneurysm; USG, ultrasound-guided; IVC, inferior vena cava; RV, right ventricle; VHD, valvular heart disease; DCMO, dilated cardiomyopathy; CHD, congenital heart disease; GB, gallbladder; ONSD, optic nerve sheath diameter; LV, left ventricle; POCUS, point-of-care ultrasound.

† , number of positive exams.

**FIGURE 5:**
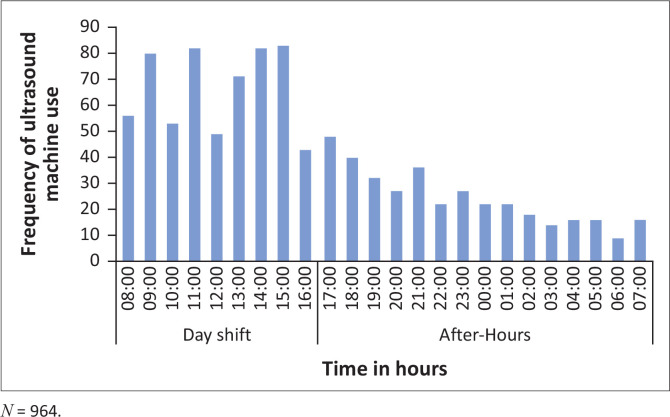
Frequency of ultrasound machine use by time of use from 01 September 2019 to 31 March 2020.

The ultrasound machine was utilised more frequently during day-shift hours as opposed to after-hour use (Figure 5).

**FIGURE 6:**
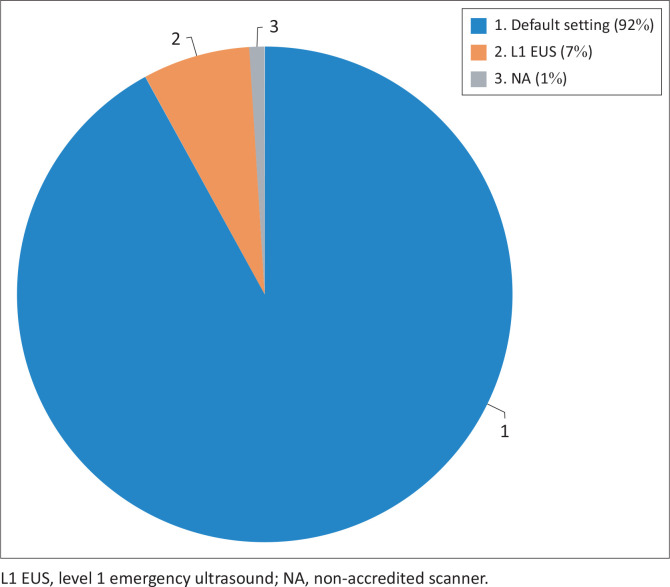
Point-of-care ultrasound scanning by level of point-of-care ultrasound accreditation.

Eight percent of POCUS data had sonographer credentials saved (Figure 6).

## Discussion

The advantages of using POCUS in the ED make it a useful tool for resuscitative and diagnostic measures, procedure guidance, therapeutic and monitoring purposes. Although 12 emergency POCUS applications have been identified as essential for practice in the ED by the ACEP, proficiency and accreditation in the five level 1 emergency POCUS exams is the minimal recommended requirement by the College of Emergency Medicine of South Africa (CEMSA) for POCUS practice within our EDs.^18,19^

Our study showed that the scope of practice of POCUS by doctors working in the ED at GJGMRH included trauma and cardiac assessment, AAA and DVT screening, first trimester pregnancy assessment, urinary and biliary tract assessment, ocular and soft tissue applications, procedure guidance and post-intubation thoracic assessment.

Focused emergency echocardiography in resuscitation and eFAST exams were the most frequent POCUS applications performed. A retrospective study by Stolz et al. reported similar indications for POCUS use in a rural ED in Uganda.^20^ In their analysis, the FAST exam was the most utilised application for POCUS (*n* = 53.3%). Interestingly, nurses were the emergency care providers performing POCUS. Potentially, task shifting of clinical skills such as POCUS may be a means of improving service delivery in rural areas where doctors and resources are very limited.

Despite the numerous benefits of POCUS, it is an operator-dependent tool that relies on the information gathered in real time during the investigation in order to aid the clinical assessment and decision-making process. Emergency ultrasound guidelines recommend POCUS documentation to include image corroboration as part of medical record.^18^ In our study, 43% of POCUS exams had incomplete imaging data to conclude, 9% had insufficient documentation of POCUS categories and 180 study participants were excluded because of absent of POCUS image data. A survey conducted by Graglia et al., identified their barriers to POCUS utility-included time constraints and discomfort with machine operation, such as storing patient data and images.^21^ A different finding was observed in a multicentre study in Malawi, Tanzania and Uganda. Shokoohi et al. identified that lack of ultrasound knowledge was the most common barrier to POCUS utility rather than time and equipment issues.^22^ Given the abrupt nature of trauma and medical presentations in the ED, delivery of emergency care takes precedence over saving ultrasound data.

The ED at GJGMRH is one of the academic sites for rotating EM registrars. To date, the department has three emergency medicine specialists. This may have contributed to the peak in POCUS utility during day-shift hours noted in our study. Multiple factors may have played a role in the decline in ultrasound use in March including the declaration of a national state of disaster as a result of the COVID-19 pandemic.

### Limitations

This study had several potential limitations. It was a retrospective review of medical records from a single source (ultrasound machine). Although the inclusion of the second ED ultrasound, patient folders and paper-based POCUS reports could have introduced conflicting data entries and data inconsistencies, exclusion of these data sources may have underrepresented the findings of POCUS utility.

Incomplete data, missing data and sonographer credentials could have influenced the accuracy of our findings. The data abstractor was not blinded to the study objectives that may have biased some study variables.

## Conclusion

The results of our study showed that the ED at GJGMRH integrated POCUS core applications for various emergency care scenarios. Further studies are needed to assess POCUS use, barriers and challenges with adopting POCUS practice by associated acute care medical specialties such as paediatric and adult intensive care units.
